# Passive sampling methods for contaminated sediments: Risk assessment and management

**DOI:** 10.1002/ieam.1511

**Published:** 2014-02-18

**Authors:** Marc S Greenberg, Peter M Chapman, Ian J Allan, Kim A Anderson, Sabine E Apitz, Chris Beegan, Todd S Bridges, Steve S Brown, John G Cargill, Megan C McCulloch, Charles A Menzie, James P Shine, Thomas F Parkerton

**Affiliations:** † USEPA Office of Superfund Remediation & Technology InnovationEdison, New Jersey; ‡ Golder Associates LtdVancouver, British Columbia, Canada; § Norwegian Institute for Water ResearchOslo, Norway; ∥ Oregon State UniversityCorvallis, Oregon, USA; # SEA Environmental Decisions LtdHertfordshire, United Kingdom; †† California State Water Resources BoardSacramento, California, USA; ‡‡ US Army Corps of Engineers, Engineer Research & Development CenterVicksburg, Mississippi; §§ The Dow Chemical Company, Spring HousePennsylvania, USA; ∥∥ Delaware Department of Natural Resources and Environmental ControlNew Castle, Delaware, USA; ## Sediment Management Work GroupDetroit, Michigan, USA; ††† Present address:The Dow Chemical CompanyMidland, Michigan, USA; ‡‡‡ Exponent IncAlexandria, Virginia, USA; §§§ Harvard University School of Public HealthBoston, Massachusetts, USA; ∥∥∥ ExxonMobil Biomedical Sciences IncHouston, Texas, USA

**Keywords:** Passive sampling methods, Bioavailability, Contaminated sediments, Risk assessment, Risk management

## Abstract

This paper details how activity-based passive sampling methods (PSMs), which provide information on bioavailability in terms of freely dissolved contaminant concentrations (*C*_free_), can be used to better inform risk management decision making at multiple points in the process of assessing and managing contaminated sediment sites. PSMs can increase certainty in site investigation and management, because *C*_free_ is a better predictor of bioavailability than total bulk sediment concentration (*C*_total_) for 4 key endpoints included in conceptual site models (benthic organism toxicity, bioaccumulation, sediment flux, and water column exposures). The use of passive sampling devices (PSDs) presents challenges with respect to representative sampling for estimating average concentrations and other metrics relevant for exposure and risk assessment. These challenges can be addressed by designing studies that account for sources of variation associated with PSMs and considering appropriate spatial scales to meet study objectives. Possible applications of PSMs include: quantifying spatial and temporal trends in bioavailable contaminants, identifying and evaluating contaminant source contributions, calibrating site-specific models, and, improving weight-of-evidence based decision frameworks. PSM data can be used to assist in delineating sediment management zones based on likelihood of exposure effects, monitor remedy effectiveness, and, evaluate risk reduction after sediment treatment, disposal, or beneficial reuse after management actions. Examples are provided illustrating why PSMs and freely dissolved contaminant concentrations (*C*_free_) should be incorporated into contaminated sediment investigations and study designs to better focus on and understand contaminant bioavailability, more accurately estimate exposure to sediment-associated contaminants, and better inform risk management decisions. Research and communication needs for encouraging broader use are discussed. *Integr Environ Assess Manag* 2014;10:224–236. © 2014 The Authors. *Integrated Environmental Assessment and Management* published by Wiley Periodicals, Inc. on behalf of SETAC.

## Editor's note

This paper represents 1 of 6 papers in the special series “Passive Sampling Methods for Contaminated Sediments,” which was generated from the SETAC Technical Workshop “Guidance on Passive Sampling Methods to Improve Management of Contaminated Sediments,” held November 2012 in Costa Mesa, California, USA. Recent advances in passive sampling methods (PSMs) offer an improvement in risk based decision making, since bioavailability of sediment contaminants can be directly quantified. Forty four experts, representing PSM developers, users, and decision makers from academia, government, and industry, convened to review the state of science to gain consensus on PSM applications in assessing and supporting management actions on contaminated sediments.

## Introduction

### Background

Considerable interest has been expressed in advancing the use of passive sampling methods (PSMs) and measurements of freely dissolved concentrations (*C*_free_) to increase awareness among the regulatory and regulated communities, as reflected in recent documents from the US Environmental Protection Agency (USEPA 2012a, 2012b, 2012c). The present paper describes how site investigations and risk assessments can use PSMs to collect information on *C*_free_ in sediment porewater to improve risk management decision-making at contaminated sediment sites. Although the focus of this paper is on applications of PSMs for organic contaminants, similar applications should be possible for inorganic contaminants (e.g., metals), once the application of PSMs to inorganic contaminants is further developed (Peijnenburg et al. this issue). The use of PSMs relative to key management questions (e.g., nature and extent of contamination, historical and ongoing sources, risk) and remedial actions (e.g., remedy design and effectiveness, risk reduction) drew from an earlier workshop on key needs for long-term management of contaminated sediment (Thompson et al. 2012) and is the focus of this manuscript along with example applications. The paper concludes with a brief discussion of future research and communication needs.

### Why use PSMs to measure *C*_free_?

Bulk sediment chemical concentrations (C_total_) have traditionally been used in contaminated sediment site characterization, risk assessment, and risk management. The limitations associated with the use of bulk chemical concentrations as Sediment Quality Guidelines have been described in Wenning et al. (2005). In particular, without an understanding of bioavailable chemical concentrations, risk assessments carry a relatively high level of uncertainty, and therefore, so also do risk management decisions and actions that depend on such assessments. For example, failure to accurately represent exposure endpoint concentrations can result in orders of magnitude errors in risk estimates (Selck et al. 2012).

The freely dissolved concentration of a contaminant in porewater (*C*_free_) is a better predictor of contaminant bioavailability than bulk sediment chemical concentrations (NRC 2003; Vinturella et al. 2004; Lydy et al. this issue). *C*_free_ is an indicator or measure of the amount of freely available contaminant that can potentially be transferred from bedded sediments into the overlying water column and which may result in direct toxicity (adverse effects), bioconcentration (uptake via water only), and bioaccumulation (uptake via water and food) (Mayer et al. this issue). *C*_free_ also can provide insights into appropriate risk management strategies and actions to address different exposure pathways and risks. Thus, *C*_free_ information provides more certainty for contaminated sediment assessment and risk management than bulk chemical analyses. To date, PSMs have been primarily used to evaluate the following chemicals in sediments: polycyclic aromatic hydrocarbons, polychlorinated biphenyls (PCBs), and chlorinated pesticides such as dichlorodiphenyltrichloroethane and related breakdown products (DDx) and chlordane.

Freely dissolved concentrations currently can be obtained in 3 ways: 1) equilibrium partitioning theory in conjunction with empirical organic carbon–water partition coefficient and octanol–water partition coefficient relationships to predict the freely dissolved contaminant concentrations in porewater; 2) extraction of porewater followed by direct measurement of contaminant concentrations; and 3) use of PSMs. Each of these methods possesses inherent uncertainties; however, PSMs have advantages over equilibrium partitioning theory because predictions of porewater concentrations from organic carbon-normalized total sediment concentrations do not account for the heterogeneous nature of potential binding phases and potential for non-equilibrium conditions (Kreitinger et al. 2007; Mayer et al. this issue).

Passive sampling methods also provide a simpler, less disruptive sampling approach than conventional porewater collection and measurement techniques, which can provide misleading information (Chapman et al. 2002).

The materials used to construct PSMs are relatively inexpensive and commercially available, and these materials can be used to detect a wide range of sediment-associated contaminants in porewater (Lydy et al. this issue; Peijnenburg et al. this issue). Their use for characterizing conditions relevant to exposure and risks depends on using sampling and deployment designs that account for the small-scale variations that exist at the scales of the samplers. Because in situ (i.e., field) measurements using PSMs can yield time-integrated concentrations, these devices are useful for measuring gradients of *C*_free_ through the vertical profile of the sediment as well as between the sediment and overlying water column. Those types of measurements can provide insights into the direction and intensity of the diffusive flux of contaminants.

Finally, using PSMs to quantify *C*_free_ can greatly assist in answering the 3 most important management questions related to contaminated sediments:

1. Do contaminated sediments pose an unacceptable risk to ecological receptors or to human health?
2. If contaminants in sediments pose an unacceptable risk to ecological receptors or to human health, how can these risks be effectively mitigated?
3. If surface water is contributing to unacceptable risk to fish, wildlife, or humans as a result of contaminants associated with point and nonpoint sources, including sediments, how can such risks be effectively managed and reduced?

## Conceptual site models

The investigation, assessment, and management of contaminated sediment sites are typically guided by using conceptual site models (CSMs). CSMs describe the processes contributing to risks at a site and are used to: develop hypotheses to be tested; organize data collection efforts; and direct the analyses, modeling, and interpretation of collected data. The process of developing and refining CSMs assists investigators and managers in identifying and understanding key physical, chemical, and biological processes that govern the exposure of contaminants to receptors. Most importantly, CSMs can be used to evaluate and select which parameters (e.g., concentration, flux) are the most appropriate indicators of exposure and risk at a site. As part of this evaluation, managers can identify where and when PSMs can be used to measure *C*_free_.

**Figure 1:**
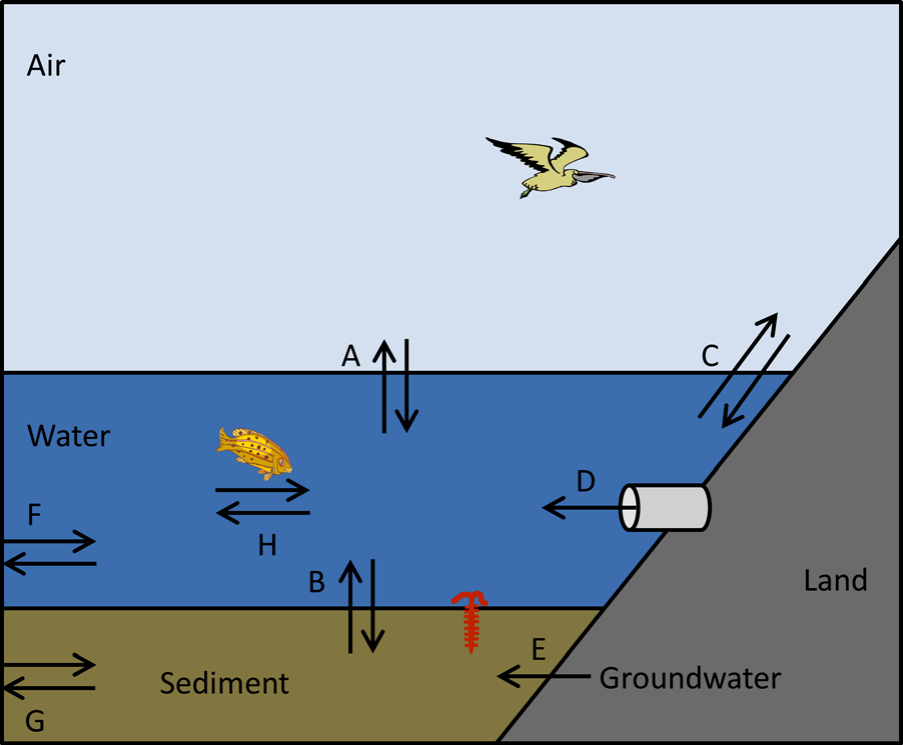
Conceptual site model showing transport and exposure pathways of sediment-associated contaminants at a macroscale. (A) Air–water exchange. Contaminant transport through air deposition into the water and volatilization from the water into the air. (B) Sediment–water flux. Diffusive and active transport processes of contaminants across the sediment–water interface, including biological processes that assist or retard transport (e.g., bioturbation, bioirrigation, development of biological secretions, biofilms). (C) Land–water exchange. Surface runoff from contaminated soil, erosion of contaminated soil particles. Deposition of contaminated water and sediment onto land during high-water or flood events. (D) Point-source inputs. Storm water discharge, combined sewer overflows, sanitary sewer overflows, industrial effluents and outfalls, spills. (E) Groundwater discharge. The discharge of groundwater contaminated from land-based sources. (F) Surface water transport. Movement of surface water containing dissolved, colloidal, and particulate-associated contaminants. (G) Sediment transport. Movement of both clean and contaminated sediment particles through bed-load and suspended-sediment transport processes. (H) Bioaccumulation. Contaminant bioaccumulation through contact with dissolved-phase contaminants and the ingestion of contaminated sediment and prey (trophic transfer).

**Figure 2:**
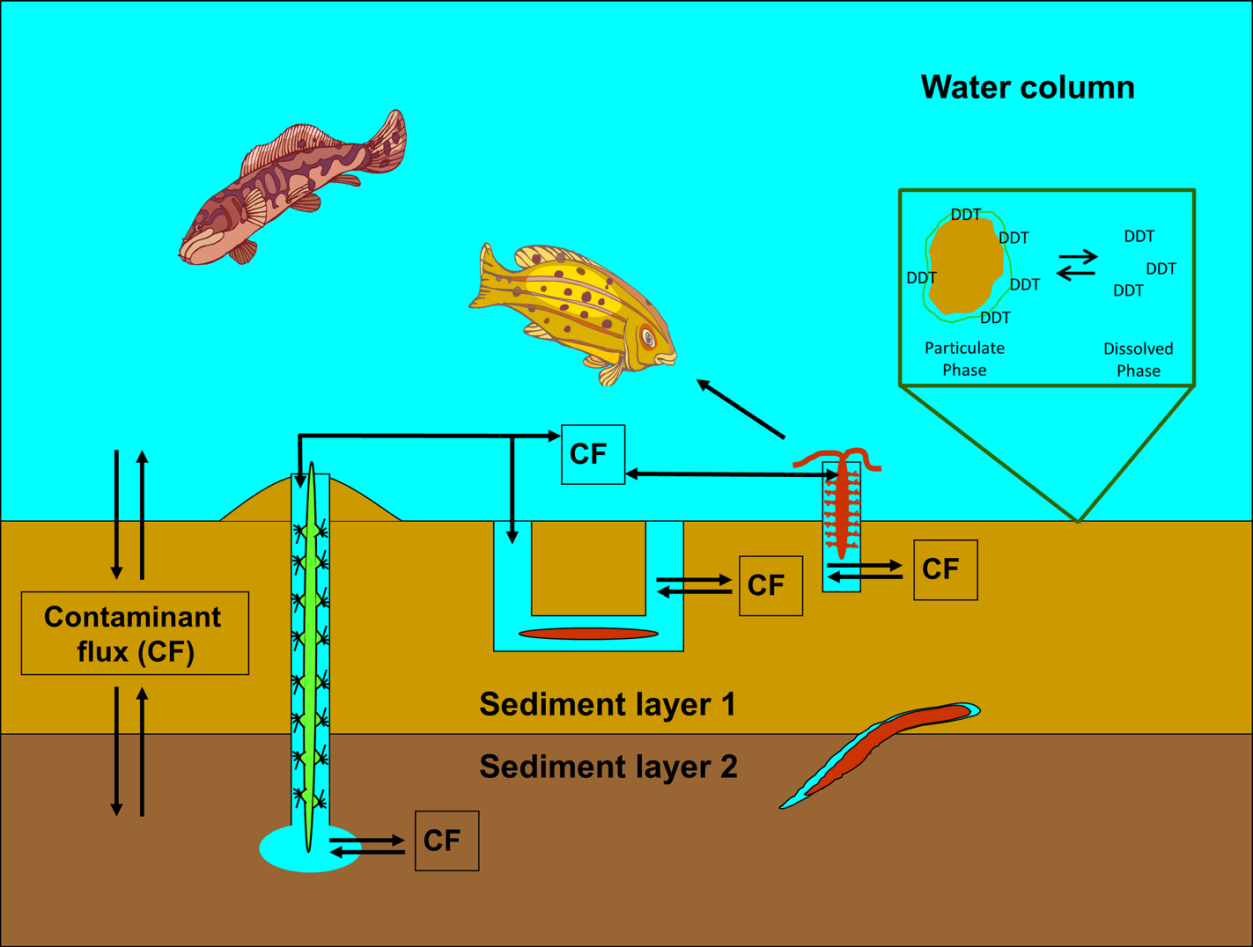
Conceptual site model showing transport and exposure pathways of sediment-associated contaminants at a microscale. Positions where contaminant flux is indicated are candidate locations for measurement of freely dissolved contaminant concentrations (*C*_free_) using passive sampling methods (PSMs).

Fate and transport of contaminants in sediments are influenced by macroscale processes that need to be retained in CSMs intended to reflect specific reaches or areas of water bodies in larger aquatic systems. At the macroscale, larger-scale hydrodynamic processes drive the transport and fate of contaminated sediments, and thus the potential redistribution, dilution, or burial of contaminated sediments. Depending on site-specific conditions and the specific objectives of a given assessment or management activity, CSMs that incorporate pathways for which *C*_free_ is a relevant metric may also need to integrate microscale exposure features with macroscale processes to provide the proper context for assessment and management. CSMs that illustrate larger-scale and smaller-scale exposure, and associated fate and transport processes, are illustrated in Figures 1 and 2. Brief examples of investigations that follow these CSMs are provided in *Programmatic Applications*.

For contaminated sediment site assessments, 4 key exposure endpoints exist for which *C*_free_ is the desired metric for risk assessment related to the 3 key management questions noted earlier:

1. Exposures of benthic invertebrates resulting in toxicity, which can result in: alterations in benthic community structure, sustainability of particular groups of species, or sustainability of a prey base for other species such as fish (i.e., “direct exposure effects”)
2. Bioaccumulation of chemicals into benthic invertebrates with subsequent exposures to fish and wildlife that feed on these invertebrates (i.e., “indirect exposure effects”)
3. Flux of freely dissolved contaminants from the sediments into the overlying water column with subsequent potential exposures of water column biota such as algae and fish directly as well as indirectly via trophic transfer (i.e., “sediment–water transport pathways”)
4. Water column exposures that reflect the net result of processes or sources, including air–water exchange, sediment–water exchange, groundwater–sediment exchange, groundwater–surface water interactions, land–water exchange, and other point and nonpoint sources illustrated in Figure 1 (i.e., “overlying water exposure pathways”)

However, risk assessors and managers need to be aware of exposure endpoints for which *C*_free_ is not an adequate metric. Examples include ingestion of sediment by foraging fish and wildlife or by children playing along shorelines or in shallow water, and direct human contact with sediment in shallow waters. Although trophic transfer pathways leading to fish that are eaten by wildlife and humans can be modeled using *C*_free_ measurements in sediments and the water column, direct measurements of fish tissue concentrations are typically required for verification of predicted tissue concentrations used in site risk assessment. Additionally, the application of PSMs to elucidate the exposure and uptake of metals by sediment-associated organisms, including dietary and incidental sediment ingestion pathways, is limited at this time (see review by Peijnenburg et al. this issue).

In addition to the macroscale processes depicted in Figure 1, PSMs can also be used to resolve the roles of processes operating at smaller spatial scales. Figure 2 illustrates key processes operating within sediments at the scale of microns to meters, including organism behaviors that affect their exposure to sediment-associated contaminants, as well as the exposures of other organisms. As with all field studies, sampling plans involving PSMs should be designed on spatial and temporal scales appropriate to address the specific study questions, as discussed in Ghosh et al. (this issue).

## Investigation/site characterization

Whereas PSMs have as yet not been widely used for supporting regulatory programs, a number of possible applications exist. The following sections discuss these potential applications.

### Design, Scale, and Temporal Considerations

Guidance on optimizing sampling designs is available (e.g., USEPA 2002a); however, specific guidance related to selection and applications of PSMs to contaminated sediments is evolving (USEPA 2012a; Ghosh et al. this issue). Assessing the extent of contamination at a sediment site can be a complex task with accompanying geographic, seasonal, financial, and cultural considerations. The key is to determine the questions that need to be answered to effectively assess and manage risks, such that targeted data collection can be effectively and usefully conducted within the limits of available resources.

Project-specific questions related to *C*_free_ include the 4 key exposure endpoints discussed previously under CSMs. Spatial and temporal scales along with horizontal and vertical heterogeneity of sediment characteristics and contaminant distributions need to be factored into the sampling design. For example, exposures of benthic invertebrates occur at small scales (Figure 2). In contrast, flux to the water column and associated water column exposures may be most relevant for larger areas or volumes inhabited by higher trophic-level organisms, including fish (Figure 1). Strategic approaches for collecting *C*_free_ information differ depending on the scale of the investigation.

Because not all transport or exposure pathways or receptors will be present at every site, use of PSMs should reflect site-specific conditions and data needs. Similarly, the extent of sediment contamination will vary across a site, with a consequent level of uncertainty dependent on the site assessment design. This uncertainty can be reduced by deploying PSMs on multiple field-collected samples or with multiple in situ deployments.

### Ambient Monitoring

The use of PSMs as monitoring tools in water is well established and is analogous to the “Mussel Watch” Program (Cantillo 2003; Smedes 2007). PSMs can be used as “artificial mussels” to monitor contaminant levels in surface waters and in sediments, including discontinuous or seasonal discharges (Laane et al. 2012). Although PSMs do not incorporate processes that can influence bioaccumulation such as dietary exposure, growth dilution, and transformation, sampling rates and equilibrium partitioning are better defined.

A PSM-based sampling program should be designed to collect data that are valid representations of the situation being assessed and not compromised by confounding factors such as pre- or postdeployment contamination or analyte losses. Sample replication and deployment sequencing sufficient to accommodate site and temporal variability, and quality assurance/quality control protocols, are also required (Ghosh et al. 2014).

In situ PSM deployments should be in locations, at depths, and at times relevant to the objectives of the sampling program. For example, relative to source(s) of sediment contamination (see next section), PSMs might be deployed along horizontal or vertical transects. Ex situ (i.e., in the laboratory) PSM applications also need to be temporally and spatially relevant. Additionally, the differences between field (in situ) and laboratory studies need to be considered. For example, differences in accumulation between laboratory exposures for field-collected sediments that have been mixed and statically deployed samplers in situ may be attributable to the deployment procedure, not necessarily because *C*_free_ is truly different. For samplers of identical surface areas, the uptake rate under static conditions in sediment has been shown to be lower than for sediments that have been mixed. Tomaszewski and Luthy (2008) found lower contaminant accumulations in PSMs under field conditions than in the laboratory, but they were able to adjust for this difference using performance reference compounds.

Because contaminants accumulated by a PSM constitute an extract from the sediment in which the PSM was deployed, some factors cause uncertainties in the rate of transfer of contaminants from the sediment. Because lower molecular weight chemicals will reach equilibrium faster than higher molecular weight chemicals, the duration of the deployment of a PSM in sediment is a factor that influences the results. In addition, the development of bacterial, fungal, or algal films on the surface of the PSM can occur over time, and these biological films would presumably affect the sorption performance of the device, thus complicating interpretation of PSM data. Although one may not always be able to obtain absolute measures, relative estimates of exposures concentrations and bioavailability can be achieved by comparing masses of contaminants accumulated by PSMs deployed under comparable conditions and for the same deployment periods (Ghosh et al. 2014).

### Source Identification (Pathways) and Quantification

Quantifying the contributions of land-based sources to sediment contamination at urban sites poses a difficult challenge because of nonpoint sources (Environment Canada 1998; USEPA 2004). Although PSMs have not been widely used for source identification or for supporting regulatory programs, a number of applications are possible. PSMs measuring *C*_free_ can be used to indicate sources and relevant exposure pathways, including those associated with bedded and resuspended sediments, as well as changes in emissions from sources and resulting exposures. PSMs also can assist in determining whether contaminants might be mobile, or to signal changes in contaminant mobility caused by changes in biophysical conditions. When deployed over relevant spatial or temporal scales, PSMs can be used to identify and characterize contaminant concentration gradients and phase distributions. These data can, in turn, be used to indicate potential sources of contaminants.

Management decisions addressing both point and nonpoint contaminant sources, such as the establishment of total maximum daily loads, are based on evaluations of the relationship of sediments to other potential sources or environmental compartments. Measurement of *C*_free_ can inform current and future roles of sediment as a source of contaminants to the surface water and biota, or as a sink for contaminants. Measurements of *C*_free_ using synoptic sampling strategies can provide information to support comprehensive assessments of multiple sources and the implications of alternative watershed management strategies, including implementation of best management practices for attaining water quality–based objectives.

Information on contaminant desorption and release from both bedded and suspended particles into the dissolved phase (i.e., *C*_free_) is critical for the development of accurate estimates of exposures at contaminated sediment sites. Quantifying the rate and total amount of contaminant released into the dissolved phase through resuspension will improve the accuracy of baseline risk assessments (see section on *Using C*_*free*_ *in multiple lines of evidence risk assessment*) and will provide critical information for informing the design of remedies (e.g., best management practices) and evaluating risks related to natural events, human activities, and remedy implementation (Bridges et al. 2010).

Characterizing or predicting exposures under current and future conditions (e.g., under baseline and alternative remedial scenarios) at a contaminated sediment site is undertaken by using information about contaminant fate and transport. Use of PSMs to measure *C*_free_ will improve characterization of these processes, and thus reduce uncertainties in site investigations and in risk analyses based solely on total mass of contaminants.

### Remedial Actions

Executing remedial actions for contaminated sediments involves a progression of activities: remedy evaluation, design, implementation, and monitoring. Designing monitored natural recovery, in situ treatment, capping, and dredging components of a site-wide remedy is undertaken by applying mechanistic information about contaminant behavior and exposure pathways to develop a strategy that will reduce long-term exposures to acceptable levels while minimizing short-term exposures resulting from management actions (USEPA 2005; Apitz 2011; Chapman and Smith 2012). Given the importance of accurately characterizing exposure in remedy design, *C*_free_ represents a critical engineering parameter. For example, because a cap needs to be designed such that exposure point concentrations in the biologically active zone of the cap do not exceed concentrations that would cause toxicity to benthic invertebrates colonizing the surface layer of the cap, *C*_free_ is required for effective cap design, because this exposure endpoint determines toxicity.

Remedy construction activities will result in the physical disturbance of sediment and overlying water. For example, dredging will resuspend sediments, resulting in the release of contaminants into the water column in particulate, colloidal, and freely dissolved (*C*_free_) form (Bridges et al. 2010). For large projects these releases, while individually short term in generation, can exist in the water column for extended periods over the course of the project. In many cases, biological monitors such as mussels have been used to assess the implications of these releases from suspended sediments. PSMs also may serve this purpose. Because mussels and PSMs provide indications of exposure over longer time frames (typically weeks to months), they may not be suitable for assessing shorter-term events (e.g., on the order of hours to a few days). Consequently, monitoring of construction activities is performed to actively manage short-term risks resulting from those activities and so that remedy implementation will not compromise the long-term objectives of the remediation project or regulatory action (USEPA 2005; Wenning et al. 2006; Chapman and Smith 2012). PSMs provide a practical means for monitoring exposures and risks during remedy implementation. However, monitoring and other investigative strategies should be designed such that the timescales requiring evaluation are compatible with PSM measurements, or that temporal issues are addressed in data interpretation. Guidelines for selecting PSMs to effectively address a particular set of investigative questions are provided in Ghosh et al. (this issue).

Remedy performance monitoring is conducted to determine whether the risk reduction objectives established for the project have been achieved, or are being achieved over time (i.e., is *C*_free_ reduced or decreasing?). In the case of dredged material disposal (whether in uncontrolled disposal sites or in controlled containment), monitoring can be carried out to provide early warning of any unanticipated releases or exposures. Both in situ and ex situ applications of PSMs can be used to measure temporal trends, or to signal changes in exposure point concentrations, relative to remedial predictions.

### Modeling

Modeling is undertaken to describe and inform investigation and remedial processes that include contaminant fate and transport, remedial design, and postremedial risk reduction monitoring (Sanchez et al. 2002; Glaser and Bridges 2007; NRC 2007; USEPA 2009). Because models play a critical role, one must consider how measurements or estimates of *C*_free_ will contribute to modeling efforts and decision making that is informed by models.

**Figure 3:**
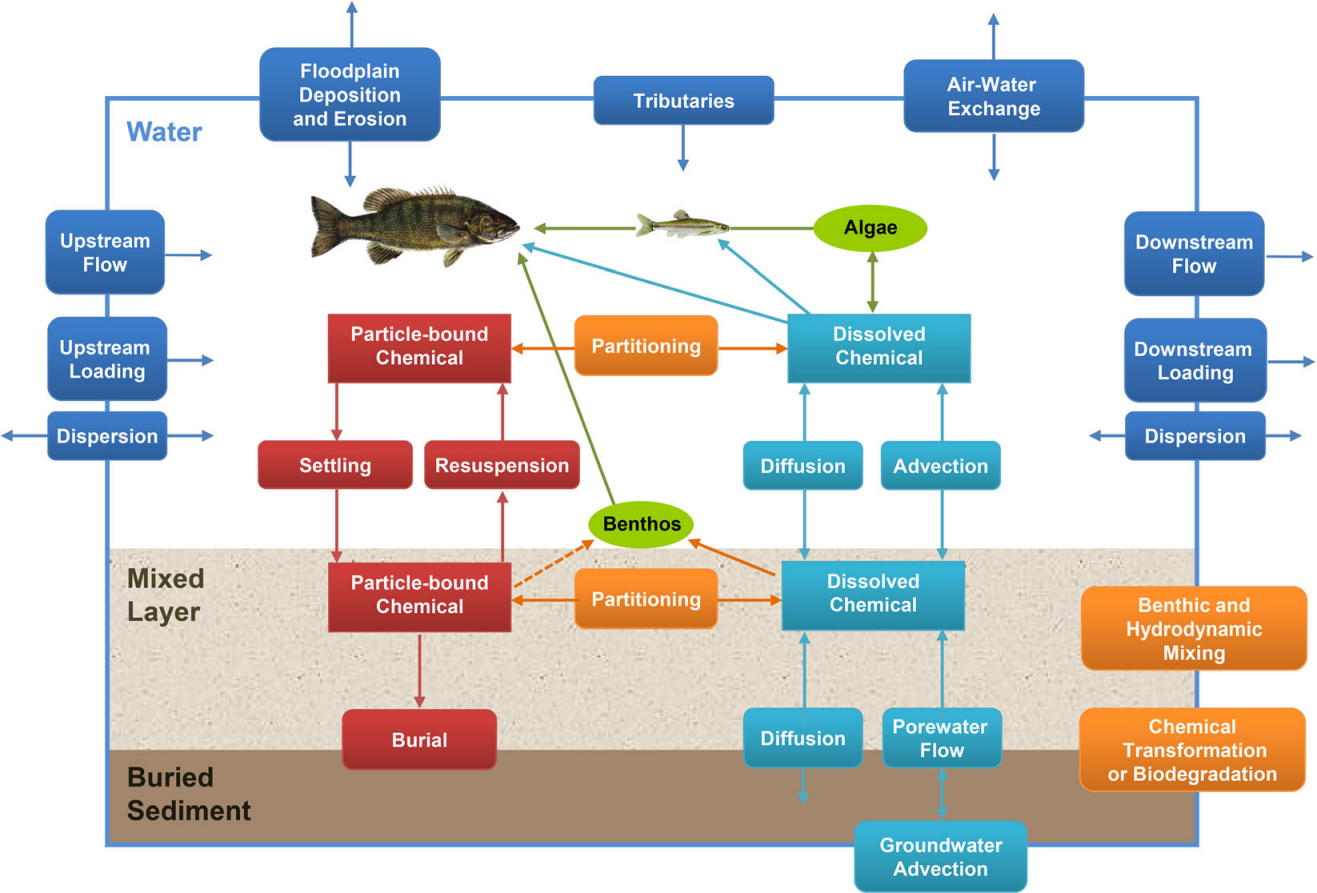
Fate and transport processes subject to modeling at contaminated sediment sites. From USEPA (2009). Estimates of freely dissolved contaminant concentrations (*C*_free_) using passive sampling methods (PSMs) are expected to reduce uncertainty in the dissolved chemical term.

Measurements that distinguish contaminants present in particulate (and colloidal) form from *C*_free_ are critical for accurately representing exposure and effect processes that may be incorporated into models. Figure 3 presents an example of fate and transport processes that are commonly represented in models applied at contaminated sediment sites. PSMs have been used in the parameterization and validation of models used to estimate the fate and risks of chemicals in contaminated sediments, particularly for nonpolar organic compounds (Vinturella et al. 2005).

Many fate and transport models start with the total concentration of an organic contaminant in the whole sediment, or C_total_, to estimate *C*_free_ in porewater. Because the freely dissolved concentration output term of partitioning models serves as an input to exposure/effects models, PSMs allow model validation or refinement.

### Using *C*_free_ in multiple lines of evidence risk assessment

Freely dissolved contaminant concentrations can be used as a line of evidence (LOE) to enhance the relevance of the chemistry data in a sediment “decision” (Maruya et al. 2012). In a weight of evidence (WOE) approach, multiple LOEs are combined using decision trees, or weighting and scoring approaches based on links between LOEs, or on the level of confidence with which different LOEs are viewed (Chapman and Hollert 2006; Apitz 2011). Bulk chemical measures such as C_total_, which ignore bioavailability, are typically given lower weightings in WOE approaches than other LOEs. *C*_free_, as either a replacement or supplemental measure to C_total_, will reduce uncertainty in the chemical LOE and thus provide a better technical basis for WOE. If monitoring programs are tiered, *C*_free_ measurements also can be used as a higher-tiered LOE to address bioavailability, thus providing for an improved understanding of potential risks. Given that *C*_free_ measurements provide estimates of chemical exposure, analyses of actual risks should also typically be supported by relevant measures to estimate possible adverse effects (e.g., organism survival, growth, and reproduction). The use of sediment toxicity testing provides a bridge between estimated chemical exposure and adverse effects, which is often used to support risk-based decision making. Benefits may arise from incorporating the use of PSMs in laboratory sediment toxicity testing of field collected sediments (i.e., to measure *C*_free_ in the exposure chambers as a dose metric). However, differences may well exist between measures of *C*_free_ in laboratory test vessels and under field conditions, and such potential differences must be understood when considering site-specific risks.

Direct measures of *C*_free_ using PSMs as part of a WOE approach using multiple LOE improves site characterization, CSM development, and tiered sediment risk assessment. At locations where PSMs are deployed, additional lines of evidence could include measurements of porewater (e.g, analysis of porewater samples obtained through centrifugation, suction, or other methods) as well as toxicity studies. The latter 2 measurements can be useful for building confidence in the *C*_free_ measurements obtained from PSMs. Additionally, vertical gradients of *C*_free_ in sediments and at the sediment–water interface can provide insights into contaminant flux. When combined with other metrics as part of a WOE analysis, measurements of *C*_free_ via PSMs provide information useful for understanding contaminant bioavailability, exposure–effects relationships, and possible chemical causes for observed toxicity.

Measurements of *C*_free_ can be used for initial screening as a separate LOE (i.e., as a PSM-based LOE for bioavailable contaminant concentrations in surface or porewater that can be screened against such benchmarks), or with other LOE in baseline risk assessments. With the cautions noted regarding potential differences between laboratory-based and field-based measures of *C*_free_ using PSMs, *C*_free_ data can be used as a dose metric to evaluate toxicity test response data relative to concentrations of contaminants that elicit toxic effects.

When exposure pathways are understood and reflected in a sound CSM, PSM data can inform and improve risk assessments involving higher trophic-level biota (wildlife and humans). As previously discussed, measured *C*_free_ concentrations in sediments and overlying water can be used as inputs to food-chain modeling. Further, PSM-derived estimates of tissue concentrations can be compared with measured tissue concentrations and tissue quality benchmarks (i.e., critical body residues) to gain insights regarding biomagnification or trophic dilution and subsequent risks, respectively (Mayer et al. this issue).

Many food-chain models begin with estimates of chemical exposure concentrations in the water column and sediment. Again, for those situations in which the exposure pathways have been properly identified and characterized, measurements of *C*_free_ via PSMs could be used to help parameterize models. Allan et al. (2011) describe the use of these devices in the context of human health risk assessment. Although the sampling of biota tissues will remain central to human health risk assessments or contaminants transferred via food webs, measurements of *C*_free_ can help inform managers concerning the potential that a site would contribute to concentrations of contaminants in biota tissues. Furthermore, the PSM data would provide a basis for assessment that is unaffected by the variability inherent in sampling in sampling biota, which often provide limited specific spatial or temporal information relative to sources of contaminants and human consumer exposures (USDOI 1998; Schwartz et al. 2005). Whereas measurements of *C*_free_ cannot substitute directly as a risk metric (because water does not behave as fish), these measurements provide insight into the potential for bioaccumulation (NRC 2003; Walker et al. [Bibr b91]).

## Management applications

To date, the measurement of *C*_free_ via PSMs has not been used consistently in regulatory programs involving contaminated sediments. This section, written from a prospective standpoint, considers the use of *C*_free_ as measured by PSMs for a range of management applications. The selection and implementation of remedies to manage unacceptable risks from contaminants in sediments and surface water in terms of the 3 key management questions detailed earlier (*Why use PSMs to measure C*_*free*_*?*) can be guided by: evaluating the relative magnitude of *C*_free_ as a source of exposure and risk, exposure pathways developed using the CSM, and risk management options for reducing risks to acceptable levels. *C*_free_ determinations can be used to map areas of potential concern, which can be linked to site remedial goals and used to support the development of remedial zones (action areas). Three-dimensional remedial zones can be defined by coupling exposure and risk factors with other site characteristics (e.g., geomorphological features, habitat types, land and waterway use, sediment characteristics, navigation requirements, presence or absence of debris) to facilitate evaluation of remedial technologies and overall risk management options.

### Remedy design and monitoring

Remedy designs are based on information that relates the behavior of risk-driving contaminants to risk-reduction objectives (e.g., cleanup levels). Data on contaminant desorption and release from particles, combined with measurements of *C*_free_, provide critical insights needed to estimate the flux of contaminants within the sediment bed, contaminant release from resuspended particles, and the magnitude of resulting exposures.

Monitoring before (i.e., baseline monitoring), during (i.e., remedial action monitoring), and after remedy implementation or pilot studies typically measures contaminants in bulk sediments, overlying water and porewater, and biota. Monitoring of remedy effectiveness is a critical aspect of contaminated sediment management (Gustavson and Greenberg 2013). A successful sediment remedy is one in which the “selected sediment chemical or biological cleanup levels have been met and maintained over time, and where all relevant risks have been reduced to acceptable levels” (USEPA 2005).

Design of in situ treatment using amendments to sequester contaminants (e.g., activated carbon) is dependent on information regarding the partitioning behavior of the target contaminants. PSMs measuring *C*_free_, deployed in situ and ex situ, are ideally suited to provide this specific information. Cap designs make use of information regarding contaminant movement, largely through porewater diffusion and advection, to determine design features that will limit flux to the surface of the cap and the overlying water. Ex situ application of PSMs as part of bench-scale column studies can provide valuable information on predicted flux and exposure in response to sediment amendments.

The effectiveness of caps depends on the creation of a clean surface through the burial and isolation of contaminated sediments and the maintenance of clean conditions through the prevention of flux or migration of contaminants (e.g., via upwelling or the advective flow of porewater). Isolation of contamination below a cap may involve chemical or physical sequestration. Monitoring the performance of a cap designed for long-term isolation or sequestration of contaminants to evaluate whether contaminant migration into the biologically active zone is present can be achieved through the use of PSMs configured to sample at specific depths in the sediment bed, to obtain a continuous depth profile (Reible et al. 2009), or placed at the sediment–water interface to gauge whether contaminants have migrated into the water column. A major advantage of using PSMs for cap performance monitoring is that sampling can be performed in situ with minimal disruption to the cap itself.

The use of PSMs as monitoring tools has been examined by a number of studies that are part of the Strategic Environmental Research and Development Program and Environmental Security Technology Certification Program (SERDP-ESTCP) to evaluate the methodology and explore the efficacy of treatment or amendments. SERDP-ESTCP projects involving PSMs have focused on baseline conditions (Gschwend 2010), in situ treatment applications (Luthy 2005; Gschwend 2009; Chadwick 2013), and capping (Thomas et al. 2012).

### Monitoring bioaccumulation by fish and shellfish

Tissue concentrations of contaminants such as PCBs and dioxins, and some metals in edible tissues, are often the risk drivers at contaminated sediment sites. Fish tissues from species targeted by anglers often receive the most attention, and many of these species are predominantly pelagic. PSMs can serve an additional purpose in the context of water column monitoring as an indicator (but not necessarily a surrogate) of contaminant bioaccumulation in edible fish and shellfish tissue. This use as an indicator reflects changes in overlying water concentrations of contaminants that can affect uptake into aquatic organisms. The combined evidence of PSMs in the surface water and porewater—the latter an indicator of bioaccumulation by sediment-associated biota that may be prey items of fish—can be used to determine when fish tissue evaluations would be appropriate for assessing whether fish consumption advisories should be set, maintained, or relaxed.

This approach reduces the need for regular (e.g., annual) destructive sampling (sacrifice) of live indigenous organisms, because confirmatory sampling of site biota would be triggered only if the PSMs indicated that a substantive change in surface water or porewater contaminant concentrations had been observed. It also reduces costs by precluding the need for biota collection, preparation, extraction, cleanup, and analyses. Seasonal or other short-term fluctuations in live specimen conditions that complicate interpretation of bioaccumulation data also can be avoided using PSMs. Furthermore, this approach assists in explaining the importance of considering other sources of contaminants to biota, including trophic transfer, when target species have relatively large home ranges relative to the contaminated sediment site under consideration (i.e., the “fish swim” conundrum).

### Evaluating sediment remediation success

Evaluating remedy success involves many of the uncertainties inherent to risk assessment, with the added complexity of temporal variability in the performance of engineered processes and features (NRC 2004). Under an adaptive management approach (Satterstrom et al. 2007), innovative alternatives found to be most effective in reducing *C*_free_, and hence contaminant bioavailability, exposure, and risks, can be incorporated into final risk management and site remedial decisions. Because the performance of many remedial approaches involves 1 or more best management practices, such as containment, treatment, and attenuation of *C*_free_, PSMs can be used to evaluate remedy effectiveness at various stages in the remedial selection and implementation process. PSMs also can be used to evaluate *C*_free_ in sediment that is slated for disposal or beneficial reuse after management actions (e.g., maintenance dredging).

PSMs can provide a reference point for assessing long-term remediation success across different remedial strategies (Chapman and Smith 2012). For example, monitored natural recovery relies on risk reduction through chemical transformation to less persistent or toxic moieties, chemical sequestration, and burial of contaminated sediments with cleaner materials through natural sedimentation processes. PSMs can be placed in the sediments in situ, or ex situ using core samples, to evaluate whether transformation processes are ongoing and whether contaminant concentrations in the porewater are changing with time.

## Programmatic applications

### Example: USEPA

Use of PSMs is increasing at USEPA Superfund and Great Lakes Legacy Act sites. Uses include revising CSMs, sediment risk assessment, and monitoring bioavailability and contaminant flux.

At the Palos Verdes Shelf Superfund site, PSMs were used in deep water to measure *C*_free_ of DDx and PCB congeners within a few meters of the sediment–water interface (Fernandez et al. 2012a). The PSMs allowed for preremedial quantitation of low dissolved concentrations of PCBs and supported the CSM, which hypothesized that contaminants in the sediments enter the water column and are bioavailable to fish. A second study employing PSMs on a benthic flux sled configuration to quantify sediment–water exchange of PCBs and DDx is in progress (Fernandez et al. 2012b). PSMs are scheduled for use in postremedial monitoring of PCBs and DDT after the targeted capping of heavily contaminated sediments (Robert Burgess, USEPA, Narragansett, RI, USA, personal communication).

At the Grasse River Superfund site, a pilot study was conducted to evaluate whether granular activated carbon placed in sediments could effectively reduce the bioavailability of PCBs to benthic organisms and flux to the water column. In situ monitoring of the sediment and water column was performed using PSMs after application of activated carbon. Substantial reductions (>90%) in contaminant bioavailability and exposures to benthic organisms were documented (Beckingham and Ghosh 2013).

Other examples of PSMs used at Superfund sites are the use of porewater peepers for metals (MacDonald et al. 2009; Brumbaugh et al. 2011) and solid-phase microextraction (Steevens et al. 2011) for PCBs, to develop concentration–response relationships in laboratory toxicity tests with benthic organisms. In situ PSMs deployed in sediments and the water column have been used to evaluate groundwater transport and exposure pathways for metals and organic contaminants within the intertidal zone of a marine waterway (Duncan et al. 2007).

### Example: US state—California

California assesses contaminated sediments based on a multiple LOE approach using bulk sediment chemistry, sediment toxicity, and benthic community structure (SWRCB 2009). If the assessment indicates impairment, additional studies are required to determine whether adverse biological effects are caused by sediment contaminants and, if so, to identify causal chemical(s). The complex contaminant mixtures in the sediments that make up a significant portion of California's highly industrialized port and harbor sediments make causation determinations difficult.

Measurements of *C*_free_ would assist in determinations of causation and also could be used to supplement or replace bulk sediment chemistry measurements. Measurements of *C*_free_ could also be applied retrospectively to historic impairment determinations to improve restoration strategies and to assist in refining total maximum daily loads implementation plans for contaminants of actual concern.

### Example: Europe—Norway

The Norwegian sediment risk assessment guidelines provide a tiered regulatory tool to identify contaminated sediment sites where remediation may be needed. Porewater contaminant concentrations are 1 of the key factors that is recognized to influence contaminant fate and movement in or out of sediment and accumulation by organisms. PSMs measuring *C*_free_ are used in these assessments in Norway (Bakke et al. 2010; Saloranta et al. 2011). Other PSM applications relevant to these guidelines are in situ or ex situ measurements of sediment–surface water fluxes from benthic chambers or box cores (Eek et al. 2010) and the case study example given later (Allan, Nilsson et al. 2012; Allan, Ruus, et al. 2012).

Because of emissions from a magnesium smelter, sediments from the Grenlandsfjord (Norway), a system of 5 connected fjords, are contaminated with polychlorinated dibenzodioxins/furans (PCDD/Fs) and other chlorinated organics such as hexachlorobenzene, octachlorostyrene (OCS), or decachlorobiphenyl (PCB209) that have resulted in a seafood consumption advisory at this location. PSMs have been applied over the last decade in this region to assist in understanding contaminant fate and distribution between water and sediments and to evaluate various management options. PSMs have been used to measure dissolved PCDD/F concentrations in overlying and sediment porewaters to evaluate concentration gradients and direction of contaminant diffusive fluxes (Cornelissen et al. 2010). Laboratory-based batch exposures with polyethylene sheets have been used to estimate sediment–porewater distribution coefficients for hexachlorobenzene, octachlorostyrene, and PCB209 in sediments from 1 of the fjords, Frierfjord. A novel PSM application was employed to estimate the impact that benthic trawling (a form of commercial fishing) would have on freely dissolved PCDD/F concentrations in bottom waters because of the resuspended sediment plume.

One key sediment management objective is to remove the advisory against consumption of seafood. The application of a thin-layer cap to a relatively wide area of the Eidangerfjord was considered as a means to reduce concentrations of chlorinated organic contaminants in edible biota (e.g., cod, crab). To evaluate this option, PSMs were used in laboratory boxcore experiments to measure diffusive fluxes of the contaminants, and to evaluate the effectiveness of various thin-layer cap designs incorporating a range of carbonaceous and mineral capping materials (Josefsson et al. 2012). This work was followed by a large-scale field study in which PSMs were used in diffusion chambers to estimate sediment-to-water fluxes of PCDD/Fs, and in the water column to assess the potential for decreases in contaminant levels in the bottom waters above capped areas. PSMs provided empirical data on the potential effectiveness of various possible remedial alternatives (Cornelissen et al. 2012).

### Example: Asia—Hong Kong

Wu et al. (2007) have developed a novel “Artificial Mussel” PSM for monitoring dissolved metals in aquatic environments, which has proved effective for assessing Cd, Cr, Cu, Pb, and Zn concentrations in coastal marine waters, providing similar data as marine mussels (Leung et al. 2008). This PSM has been successfully used in Australia, China, Portugal, South Africa, the United Kingdom, and the United States (Leung et al. 2008; Degger et al. 2011; Gonzalez-Rey et al. 2011; Kibria et al. 2012). A “Global Artificial Mussel Watch” program has been launched, including deployments in Africa, Asia, America, and Australia. In addition, semipermeable membrane devices have been successfully applied to monitor polycyclic aromatic hydrocarbons, petroleum hydrocarbons, polychlorinated biphenyls, and chlorinated pesticides in the coastal marine waters of Hong Kong (Richardson et al. 2001, 2003, 2008).

## Future applications and research needs

### Metals

Additional development work is required in the use of PSMs for metals within a modeling or decision-making framework (Peijnenburg et al. 2014). Diffusive gradients in thin-film (DGT) PSMs used to examine metal bioavailability in sediments (Viollier et al. 2003) have “not been sufficiently demonstrated for sediments” (Costello et al. 2012). Research is needed to understand what species of metals and organo-metallic compounds (e.g., methylmercury, organo-tins, organo-arsenicals, selenomethionine) the DGTs measure and their relationship with toxicity (Twiss and Moffett 2002; Teuchies et al. 2012).

### Biogeochemical processes

Current PSMs used to measure the bioavailable *C*_free_ fraction in porewater provide no insights into biogeochemical processes that can affect the *C*_free_ fraction in porewater. Changes in *C*_free_ may be attributable to either changes in the biogeochemical cycling of a contaminant in the sediment or changes in the contaminant total concentration. Pairing of PSMs with desorption measurements may assist in further defining biogeochemical processes or mechanisms leading to relevant porewater exposure conditions. This information would provide risk managers with further understanding of the “sediment source” term for contaminants, which would further inform decisions regarding remedy selection and design.

### Contaminant mixtures

Identification of chemicals causing toxicity within mixtures of contaminants in sediments can be done using an effects-directed approach or toxicity identification evaluation (Reichenberg and Mayer 2006); both methods have limitations (Burgess et al. 2013). Effects-directed approach extraction methods “ignore bioavailability and thus produce a bias” and do not provide a mechanistic understanding of bioavailability (Judson et al. 2010). PSMs, deployed and allowed to equilibrate in sediments, can be used as a passive dosing source to recreate *C*_free_ exposure in ex situ toxicity tests that are performed in support of sediment toxicity identification evaluation (Bandow et al. 2009; Perron et al. 2009; Brack 2011).

### Emerging contaminants

Conventional sampling methods may overlook contaminants with unknown toxicological relevance (Brack et al. 2009). PSMs can be designed to extract a wide range of contaminants from sediments, including emerging contaminants (Booij et al. 2006; Sethajintanin and Anderson 2006; Gong et al. 2008; Li et al. 2009). Ideally future PSMs will provide a multipurpose device that is capable of collecting data on multiple contaminants of interest. The extraction process and analyses also will need to be optimized for more polar compounds.

### Integration with molecular and bioanalytical technology

Although substantial resources have been devoted to post-omics tool development, how these tools can be used to assess exposures through integration with real exposure scenarios is still a significant topic of discussion. PSMs could, in the future, be integrated into cell-based ‘omic' tools and other high-throughput bioassays, possibly as a separate approach that could be termed “partitionomics” (Phillip Mayer, Department of Environmental Science, Aarhus University, Roskilde, Denmark, personal communication). In addition, cross-linking an estrogen receptor to a solid-phase support to probe for estrogenic substances in sediments that could affect resident biota appears technically feasible. Similar tools have been developed for biomedical research (Sanghivi et al. 2011).

### Remote sensing networks

Autonomous “lander systems” such as the sediment profile image camera are used to carry: particle samplers benthic flow-chambers conductivity, temperature, and depth rosettes; or in situ porewater samplers to the sediment–water interface. PSMs can be integrated into in situ deployment systems incorporating other LOE such as bulk chemistry and toxicity (Burton et al. 2012; Rosen et al. 2012). Multisensor stations can provide complete, continuous data sets. In the Baltic and the North Sea, a network of such devices (MARNET) delivers comparable data from different regions (http://www.bsh.de/en/Marine_data/Observations/MARNET_monitoring_network/MARNET_en.jsp) and therefore serves as a tool for inter-calibration and quality control of monitoring programs. These devices are equipped with sensors for collecting basic oceanographic data and can be equipped with specific sensors such as PSMs in water and on the sediment–water interface. Such continuous deployment would improve trend analyses by collecting data of high or medium temporal resolution. Continuous deployment would also provide information on short-term changes to the bioavailability of sediment-associated contaminants relative to changes in environmental conditions, such as “aging” (i.e., changing bioavailability), climate change, and ocean acidification. Ocean acidification has the potential to significantly increase toxicity from contaminated sediments as determined in experiments measuring the flux of metals under different ocean acidities using DGTs together with toxicity tests (Roberts et al. 2013).

## Communication

Communication is essential to stakeholder (e.g., regulators, responsible parties, consultants, different governmental units, tribal or Aboriginal communities, community organizations, neighbors) acceptance of PSMs for assisting in assessing and managing risks at contaminated sediment sites (NRC 2001; USEPA 2002b, 2005; ITRC 2011; Bridges et al. 2012). Different stakeholders are likely to have different perceptions of PSMs and concerns about their use or applicability. These perceptions must be acknowledged and addressed by effectively communicating with stakeholders the advantages, limitations, uncertainties, and appropriate uses of PSMs (see also Ghosh et al. this issue).

### Key information to provide confidence in PSMs

Stakeholder confidence in, and thus, adoption of PSMs for use at contaminated sediment sites, requires effective communication of PSM salience, credibility, and legitimacy. PSMs are salient at contaminated sediment sites, as they may be used at spatio-temporal scales applicable to sediment sites (Figures 1 and 2). The credibility of PSMs is supported by the papers from the current SETAC Technical Workshop (Lydy et al. this issue; Peijnenburg et al. this issue; Mayer et al. this issue; Ghosh et al. this issue; present paper), which summarize numerous technical publications over the last 25 years. Their legitimacy is demonstrated by interlaboratory comparisons (e.g., Miège et al. 2012; Lydy et al. this issue).

In addition, the major advantages and disadvantages of PSMs need to be effectively communicated. With effective communication, the use of PSMs may facilitate increased implementation of science-based, risk-based approaches as opposed to precautionary principle-influenced sediment management decisions that do not yield appreciable benefit to human health or the environment.

### Actions required to increase confidence in PSMs

Five significant actions must occur to increase confidence in and thus encourage the use of PSMs:

1. Key information about PSMs and their applicability to sediment sites, such as those described previously, must be made readily accessible to potential users (e.g., regulators, responsible parties). That is, a bridge from the research and development literature on PSMs to practical applications of PSMs at sediment sites for potential users must be developed.
2. Based on the principles delineated in Ghosh et al. (this issue), guidance documents and operating manuals for users should be developed and issued by appropriate regulatory authorities.
3. Training for users should be developed and offered.
4. Successful applications or case studies on the uses of PSMs at contaminated sediment sites should be presented at well-attended conferences and meetings and disseminated through other outlets (e.g., webinars).
5. Key stakeholders at specific sites where applications of PSMs are being considered should be engaged in communication on issues outlined in this paper. This engagement should occur early at a particular site to increase understanding of PSMs and promote acceptance and limitations of their appropriate use. Engaging stakeholders can be more difficult if the subject of the engagement is viewed as a fait accompli.

Examples of specific actions that would increase confidence in PSMs are provided in the following paragraphs (some programmatic examples of applications are provided in *Programmatic Applications*).

The USEPA Office of Superfund Remediation and Technology Innovation and the USEPA Office of Research and Development have issued a Sediment Assessment and Monitoring Sheet (SAMS) on the use of PSMs at contaminated sediment sites (USEPA 2012a, 2012b). SAMS are technical bulletins designed to assist project managers (both government and industry) in understanding and adopting key tools for use at contaminated sediment sites. The SAMS, as well as other regulatory and guidance documents applicable to contaminated sediment sites, are available at: http://www.epa.gov/superfund/health/conmedia/sediment/documents.htm (last visited 6 December 2013). The USEPA Office of Superfund Remediation and Technology Innovation could conduct public education or training sessions on PSMs through its webinar platform, Contaminated Site Clean-Up Information (Clu-In: http://www.clu-in.org/; last visited 6 December 2013). Clu-In webinars are well publicized through USEPA's and others' listservs and websites, are easy to register for, are recorded for future availability, and can therefore reach a broad audience, which typically includes federal and state regulators, responsible parties, consultants, and attorneys.

The US Army Corps of Engineers Engineer Research Development Center should be encouraged to issue a Dredging Operations and Environmental Research Technical Note on the use of PSMs at contaminated sediment sites (http://el.erdc.usace.army.mil/dots/doer/pubs.cfm?Topic=TechNote&Code=doer; last visited 06 December 2013). Dredging Operations and Environmental Research technical notes serve as useful references and authoritative guides in addressing sediment management issues. The SERDP and ESTCP Programs have supported over 20 research projects and held numerous workshops in recent years related to PSMs (see http://serdp-estcp.org/ for specific reports). Additional technical guidance could be developed through the SERDP-ESTCP environmental research programs that would be available to a wide audience of practitioners and environmental managers.

The US Interstate Technology and Regulatory Council (ITRC) should be encouraged to develop a guidance document on the use of PSMs and hold training sessions, as has been done for their bioavailability guidance (ITRC 2011).

SedNet (http://www.sednet.org/), the European network on sediment, which provides a network for communication of key sediment topics and holds regular meetings on a range of assessment and management approaches and issues, should develop and communicate case studies using PSMs. Regulatory avenues in the European Union currently exist for PSM implementation, for instance, as part of the Water Framework Directive, but such avenues vary among member nations and sites. Workshops could be sponsored by institutions such as the European Chemicals Agency to provide further guidance.

## Conclusions and recommendations

1. Contaminant bioavailability is a central issue in sediment risk assessment and management, and potential exposure to sediment-associated contaminants is best characterized and estimated by focusing on *C*_free_ in sediment porewater.
2. PSMs provide managers with a tool needed to determine *C*_free_.
3. Use of PSMs can better inform site characterization and investigation as well as remedial actions; PSMs are increasingly being incorporated into contaminated sediment management programs.
4. Presently, direct application of PSMs is best suited to legacy, nonpolar organic contaminants; however, future applications are possible for metals, emerging contaminants, and mixtures—with potential extension to direct measurement in biota.
5. Further adoption of PSMs in contaminated sediment decision-making will require effective communication to increase confidence among stakeholders and encourage consistent application by practitioners.
6. Although regulatory applications of PSMs have been limited, the present and accompanying workshop papers in this series are expected to provide the technical basis and practical guidance needed to support increased application.
